# *In situ* bending of an Au nanowire monitored by micro Laue diffraction

**DOI:** 10.1107/S1600576715001107

**Published:** 2015-01-30

**Authors:** Cédric Leclere, Thomas W. Cornelius, Zhe Ren, Anton Davydok, Jean-Sébastien Micha, Odile Robach, Gunther Richter, Laurent Belliard, Olivier Thomas

**Affiliations:** a Aix-Marseille Université, CNRS, Université de Toulon, IM2NP UMR 7334, 13397 Marseille, France; b CEA, INAC, SP2M/NRS, 17 rue des Martyrs, 38054 Grenoble, France; c Max Plank Institute for Intelligent Systems, Heisenbergstrasse 3, 70569 Stuttgart, Germany; d Université Pierre et Marie Curie, CNRS, Institut des Nanosciences de Paris UMR7588, 4 place Jussieu, 75005 Paris, France

**Keywords:** micro-beam Laue diffraction, nanomechanical testing, Au nanowires

## Abstract

This article^1^ reports on the first successful combination of micro Laue (µLaue) diffraction with an atomic force microscope for *in situ* nanomechanical tests of individual nanostructures. *In situ* three-point bending on self-suspended gold nanowires was performed on the BM32 beamline at the ESRF using a specially designed atomic force microscope. During the bending process of the self-suspended wire, the evolution of µLaue diffraction patterns was monitored, allowing for extraction of the bending angle of the nanowire. This bending compares well with finite element analysis taking into account elastic constant bulk values and geometric nonlinearities. This novel experimental setup opens promising perspectives for studying mechanical properties at the nanoscale.

## Abstract 2

The *in situ* three-point bending of a single self-suspended Au nanowire is visualized by micro Laue diffraction. The nanowire deflection is inferred from the displacement of Laue spots and it is well described by finite element analysis taking into account geometric nonlinearities and the elastic constants of bulk Au.

## 1. Introduction   

In recent decades, nanomaterials have attracted enormous attention owing to both the ongoing miniaturization of electronic devices and the extraordinary properties of nano­structures as compared to their bulk counterparts. In particular, quasi-one-dimensional nano-objects such as nanowires, nanorods and nanotubes have attracted considerable interest as building blocks of nanosensors, nanoactuators and nanoresonators because of their unique electronic and mechanical properties (Li *et al.*, 2006 ▶). Nowadays, mechanical deformations of nanowires such as bending or elastic resonance allow us to control quantum states, paving the way for novel nanowire-based devices in the fields of spintronics and photonics (Shekhter *et al.*, 2013 ▶; Yeo *et al.*, 2014 ▶; Treutlein, 2014 ▶). With the aim of fabricating future reliable hybrid mechanical systems, a thorough understanding of the mechanical behaviour at the nanoscale remains essential.

About half a century ago, Brenner and co-workers noticed a significant evolution of the mechanical properties of nanowhiskers compared to bulk material (Brenner, 1957 ▶, 1959 ▶). With the advent of focused ion beam (FIB) microscopes, it became possible to machine out of bulk material dedicated structures for micro-mechanical tests, which revealed a clear increase of the yield strength with decreasing diameter (Uchic *et al.*, 2004 ▶; Greer & Nix, 2006 ▶; Volkert & Lilleodden, 2006 ▶; Kiener & Minor, 2011 ▶). This trend became known in the literature as ‘smaller is stronger’. However, micropillars that were not FIB machined did not show this trend but exhibited strengths close to the ultimate value of the material (Bei *et al.*, 2007 ▶), which was also reported for nominally defect free nanowires (Wu *et al.*, 2005 ▶; Richter *et al.*, 2009 ▶). With respect to elasticity, Young’s modulus for ZnO and for Ag nanowires was reported to increase with decreasing diameter (Jing *et al.*, 2006 ▶; Chen *et al.*, 2006 ▶). Moreover, anharmonic elastic behaviour has been demonstrated for tensile tested Pd nanowires (Chen *et al.*, 2012 ▶). Despite these numerous studies, the mechanical behaviour on the micro- and the nanoscale is far from being fully understood. The influence of free surfaces where defects may annihilate or which may act as nucleation sites for dislocations as well as the crystalline structure and inner grain boundaries are important aspects concerning the mechanical behaviour and the evolution of strain and defects.

In order to shed more light on these aspects, great efforts are being undertaken to realize *in situ* experiments which allow for visualizing the elastic deformation as well as the nucleation and propagation of structural defects in individual nanostructures during mechanical loading. Thanks to their high sensitivity to strain and defects, X-ray diffraction methods as well as transmission electron microscopy (TEM) are predestined techniques for such *in situ* approaches. When thinning is not required to obtain an electron-transparent specimen, TEM methods are very efficient (Minor *et al.*, 2001 ▶; Oh *et al.*, 2009 ▶; Kiener *et al.*, 2011 ▶). *In situ* TEM studies demonstrated reversible phase transitions for Ni nanowires strained up to 34.6%, which is far above typical elastic limits (Wang *et al.*, 2013 ▶). Moreover, *in situ* mechanical tests on Au nanowires revealed reversible plasticity through twinning during the tensile deformation and a de-twinning during subsequent compression (Lee *et al.*, 2014 ▶). With respect to X-ray studies on single nanostructures, *in situ* mechanical tests in combination with sub-micrometre-focused X-ray diffraction methods have great potential to investigate the alterations of the atomic lattice that are induced by the mechanical loading. Enormous progress has been made at third-generation synchrotron sources within the past two decades, and nowadays, thanks to Kirkpatrick–Baez (KB) mirrors or Fresnel zone plate lenses, synchrotron X-ray beams are routinely focused down to a few hundreds of nanometres, rendering it possible to illuminate individual nanostructures and, thus, to study their structural properties. For instance, the elastic and plastic deformations of micrometric SiGe and Cu islands have been studied *in situ*, combining a specially developed atomic force microscope (AFM) with coherent microfocused X-ray diffraction (Rodrigues *et al.*, 2008 ▶; Scheler *et al.*, 2009 ▶; Cornelius *et al.*, 2012 ▶; Beutier *et al.*, 2013 ▶). While monochromatic diffraction gives access to individual Bragg reflections in high resolution, methods based on polychromatic beams allow for measuring many diffraction peaks at the same time without any *a priori* knowledge about the crystallographic unit-cell orientation of the sample under investigation. The first *in situ* combination of mechanical test and micro Laue (µLaue) experiments was reported on micrometre-sized structures using a micro-indenter, permitting the identification of the slip system activated during mechanical loading and the determination of the density of geometrically necessary dislocations stored in the deformed material (Maaß *et al.*, 2009 ▶; Kirchlechner *et al.*, 2012 ▶; Marichal *et al.*, 2013 ▶).

The present work focuses on a new experimental approach, which closes the gap between TEM-related techniques focusing on electron-transparent structures and existing *in situ* X-ray diffraction methods that allow for studying micrometre-sized objects. We report the first *in situ* three-point bending tests on individual Au nanowires by combining µLaue diffraction and a recently developed compact scanning force microscope for *in situ* nanofocused X-ray diffraction (SFINX) studies (Ren *et al.*, 2014 ▶; Cornelius *et al.*, 2014 ▶). Here, SFINX is employed for bending a self-suspended gold nanowire whose deflection is inferred from the displacement of the Laue spots on the detector. The mechanical behaviour is well reproduced by finite element analysis (FEA) simulations taking into account the elastic constants of bulk Au as well as geometric nonlinearities. In the context of mechanical studies, this *in situ* method pushes the well established µLaue technique from the micro- down to the nanoscale.

## 2. Experimental   

**Figure 1:**
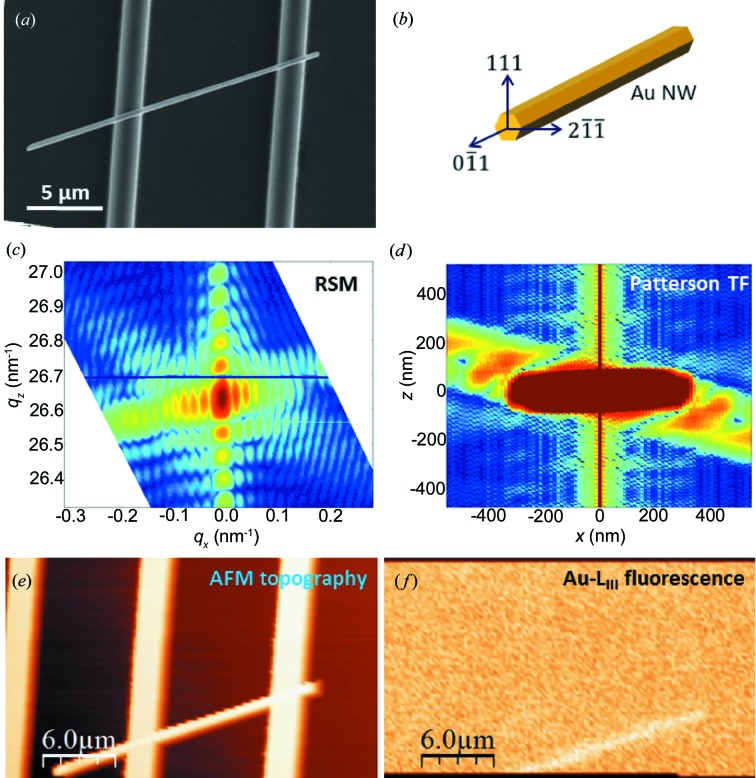
(*a*) Scanning electron micrograph of a self-suspended Au nanowire crossing an Si microtrench. (*b*) Schematic representation of the crystallographic directions with respect to the experimental configuration. (*c*) Reciprocal space map of the Au111 Bragg reflection and (*d*) the corresponding Patterson function for the Au nanowire presented in (*a*). (*e*) *In situ* AFM topography and (*f*) Au *L*_III_ fluorescence map of the Au nanowire recorded simultaneously.

Single-crystalline gold nanowires were grown by physical vapour deposition on carbon-coated tungsten substrates under ultra-high vacuum conditions at elevated temperatures as described elsewhere (Richter *et al.*, 2009 ▶). For the three-point bending tests, Au nanowires were placed across 10 µm-wide and 1.5 µm-deep microtrenches, forming self-suspended nanobridges (Fig. 1 ▶ *a*). The microtrenches were fabricated on Si(001) wafers by a combination of UV lithography and reactive ion etching. In order to avoid any sliding of the wire during mechanical testing, the wires were thoroughly clamped at the two supports by electron-beam-induced deposition of carbon from the residual gas in the scanning electron microscope chamber. The crystallographic directions with respect to the experimental configuration are depicted schematically in Fig. 1 ▶(*b*). While the growth direction of the Au nanowire is 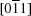 and the perpendicular direction 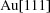 is parallel to the Si substrate normal, 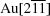 defines the bending axis and is orthogonal to the two aforementioned directions.

One of the most significant sources of persisting error in nanomechanics lies in the uncertainty of shape and size. In order to overcome these shortcomings, nanowires were investigated by coherent X-ray diffraction using microfocused X-ray beams at the CRISTAL beamline at SOLEIL. The incident X-ray beam was monochromated to a photon energy of 8.5 keV by the Si(111) double-crystal monochromator and focused down to 2 × 2 µm in the horizontal and vertical directions using a Fresnel zone plate which was installed 200 mm upstream of the sample position. A couple of high-precision slits were mounted right in front of the focusing optics set to the aperture matching the lateral coherence lengths of the incoming X-ray beam (20 µm horizontally and 80 µm vertically). The diffracted X-ray beam was recorded by a MAXIPIX pixel detector (516 × 516 pixels) with a pixel size of 55 × 55 µm mounted 1.3 m from the sample.

For *in situ* mechanical testing of a single gold nanowire in combination with Laue diffraction, the *in situ* scanning atomic force microscope SFINX was installed at the French CRG beamline at the exit of the bending magnet BM32 at the ESRF in Grenoble. SFINX is a compact atomic force microscope, which is compatible with various synchrotron endstations and thus can be combined with different sub-micrometre-focused X-ray techniques. It allows for both *in situ* imaging and *in situ* mechanical loading of individual nanostructures. Further details of SFINX can be found elsewhere (Ren *et al.*, 2014 ▶; Cornelius *et al.*, 2014 ▶). The sample placed on the specimen holder of SFINX forms an angle of 40° with the incident polychromatic X-ray beam which covers an energy range of 5–25 keV. The X-ray beam was focused down to 400 × 500 nm in the vertical and horizontal directions, respectively, using KB mirrors (Ulrich *et al.*, 2011 ▶). The diffracted X-rays were recorded by a Mar CCD (MAR165) consisting of 2048 × 2048 pixels with a size of 80 µm. It was mounted in top reflection geometry at 90° with respect to the incident beam at a distance of 70 mm, thus covering a solid angle of X-ray collection of around 100°. X-ray fluorescence excited by the focused beam was monitored using an energy-dispersive point detector (Rontec XFlash 1001, 3.4 mm opening, 10 mm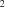 area). In addition to the three-point bending experiment, the nanowire deformation was simulated by FEA using the *COMSOL Multiphysics* software (http://www.uk.comsol.com/), taking into account the complete elastic stiffness tensor 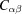 for bulk Au.

## 3. Results and discussion   

The exact shape of the self-suspended nanowire presented in Fig. 1 ▶(*a*) was determined by coherent microfocused X-ray diffraction. The cross section of the wire does not exhibit an equilibrium Wulff shape but is rather caused by the dynamics of the growth process through different growth rates of the crystal facets. A coherent X-ray diffraction pattern of the Au111 Bragg peak is displayed in Fig. 1 ▶(*c*) revealing well defined size fringes. While the centrosymmetry of the diffraction pattern demonstrates that the wire is nominally strain free, the different widths of the streaks indicate different lengths of the nanowire side facets. The exact shape of the nanowire was inferred from the Patterson function presented in Fig. 1 ▶(*d*), revealing a flattened hexagonal wire cross section with a thickness of 90 nm and a width of 360 nm.

The mechanical behaviour of this self-suspended Au nanobridge was investigated *in situ* by µLaue diffraction using the AFM. For these *in situ* studies, the AFM tip, the nano­structure and the sub-micrometre-focused X-ray beam had to be aligned with respect to each other. A first coarse arrangement was achieved by optical microscopy, allowing for positioning of the AFM tip and the selected nanowire with respect to the focal position of the X-ray beam with an accuracy of ∼10 µm. To improve the mutual alignment, the sample was scanned using the *xy* scanners of SFINX while the sample topography was recorded with the AFM tip and, simultaneously, the Au *L*_III_ fluorescence yield was monitored. Figs. 1 ▶(*e*) and 1 ▶(*f*) display, respectively, the AFM topography and the Au *L*_III_ fluorescence map for the single Au nanowire crossing the Si microtrench. The discrepancy between the two *in situ* images originates from a misalignment of the AFM tip with respect to the X-ray beam. The offset was then compensated by moving the long-range piezo motors within SFINX, eventually allowing for a perfect adjustment between the two probes: the AFM tip and the focused X-ray beam (Ren *et al.*, 2014 ▶).

**Figure 2:**
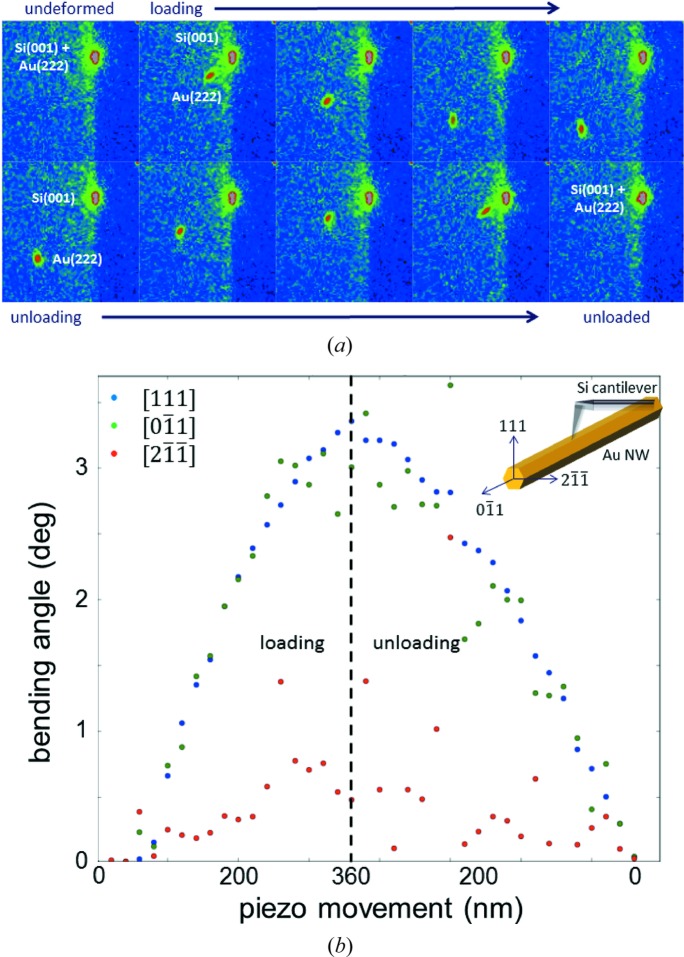
(*a*) Sequence of *in situ* µLaue diffraction patterns displaying the evolution of the Au111 and the Si001 Laue spots during the three-point bending of the Au nanowire. (*b*) Bending angle inferred from the *in situ* µLaue diffraction patterns as a function of the movement of the piezo stage carrying the AFM cantilever.

For three-point bending tests of the nanowire, the AFM tip was positioned above the centre of the self-suspended nanowire. The focused X-ray beam was displaced by 1.8 µm along the wire in order to probe a region sensitive to the bending and rotation of the wire, giving access to the orientation of the crystal. The AFM tip was lowered with a constant speed of 5 nm s^−1^, pressing against the nanowire and, hence, deflecting the self-suspended structure. Simultaneously to the bending process, µLaue diffraction patterns were recorded with an exposure time of 1 s, thus averaging over a piezo movement of 5 nm. Because of the readout time of the CCD and the time necessary for saving the file, a diffraction pattern was taken every 18.6 nm of displacement of the piezo stage. Fig. 2 ▶(*a*) presents a sequence of diffraction patterns recorded during the mechanical testing, where for simplification exclusively the evolution of the Au111 and the Si001 Laue spots are shown. Movies presenting the evolution of the complete *in situ* diffraction patterns as well as the zoom on the two central Laue spots (Si001 and Au111) can be found in the supporting material.^2^ While the two spots are superimposed for the pristine wire, the Au111 Laue spot separates from the Si001 Laue spot during the bending and moves further away with increasing load. During the unloading process the Au111 Laue spot returns towards its original position. This reversible displacement of the gold Laue spot shows a purely elastic deformation of the wire which was verified both by µLaue diffraction mapping of the whole wire after the *in situ* experiment and by scanning electron microscopy studies (not shown here). None of these later studies indicated the presence of plastic deformation or any deviation of the self-suspended wire from its original horizontal position.

From the Laue diffraction patterns the orientation, *i.e.* both bending and rotation of the wire part illuminated by the X-ray beam, was determined from the orientation matrices (UB). Taking into account at least eight Au Laue spots, the standard Laue pattern analyses were performed using the *LaueTools* software (Micha, 2014 ▶). The bending angle Θ is inferred from the dot product between crystallographic directions under load and their initial state as follows: 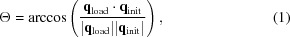
with the scattering vector 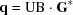, where 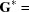 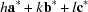 is the reciprocal-lattice vector defined with Miller indices *hkl*.

The bending angles determined for three distinct orthogonal crystalline directions as a function of the piezo movement are presented in Fig. 2 ▶(*b*). Note that the piezo movement consists of the bending of the nanowire as well as the deflection of the AFM cantilever. The inset in Fig. 2 ▶(*b*) provides an illustration of the three orthogonal crystalline directions of the Au nanowire as already depicted in Fig. 1 ▶(*b*). Au111 and 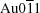 show similar behaviours during loading and unloading. The bending angle increases continuously up to 3.5° for the highest load and returns to 0° for the unloaded wire. In contrast, 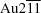 exhibits a much smaller bending in the range of half a degree only. For a perfect vertically applied mechanical load along the Au[111] direction, Au111 and 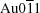 are expected to show similar behaviours, whereas 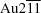 must remain unchanged. The experimentally observed bending angle along 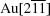 of 0.5° may originate both from the fact that the wire spans the trench diagonally and from nonvertical components of the applied mechanical load due to the deflection of the AFM cantilever.

For further analysis of the experimental data, FEA has been carried out to compute the wire deformation, taking into account the nanowire cross section obtained by coherent X-ray diffraction and the length of the self-suspended nanobridge determined by scanning electron microscopy. We used the complete stiffness tensor 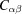 considering the values for bulk Au (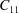 = 192 GPa, 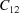 = 163 GPa and 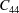 = 42 GPa) found in the literature (Neighbours & Alers, 1958 ▶). Fixed boundary conditions were applied between the wire and the support, simulating a thoroughly clamped nanobridge, and a point load was applied on the centre of the self-suspended part. In addition, the simulations were computed with and without taking into account geometric nonlinearities that originate from inhomogeneous strain fields induced by large deformations of the nanowire (Becker, 2000 ▶).

**Figure 3:**
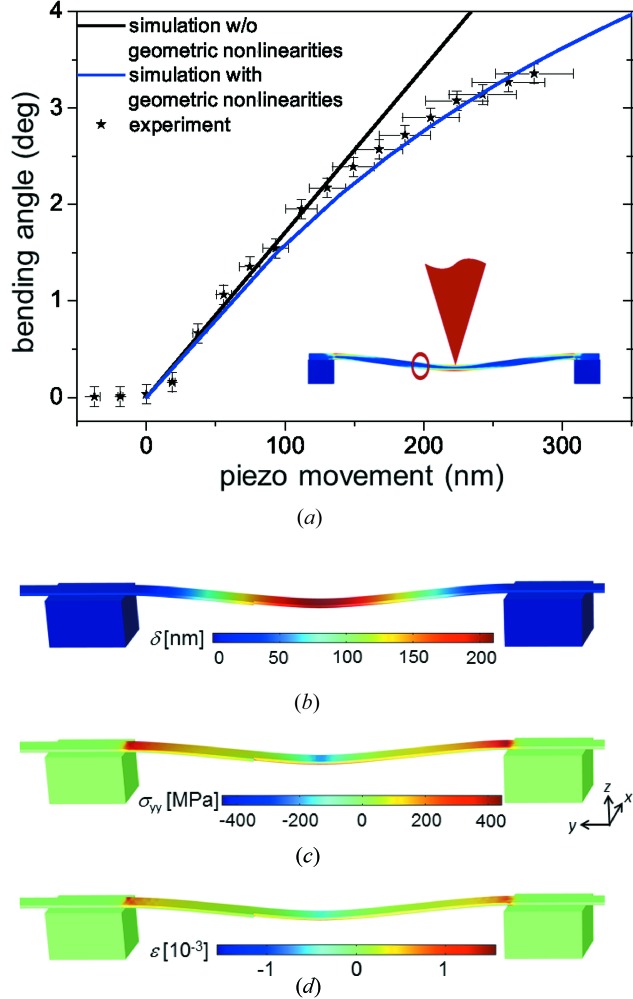
(*a*) Experimental and FEM simulated bending angle (with and without taking into account geometric nonlinearities) of the Au nanowire at 1.8 µm distance from the loading point as a function of the piezo movement corresponding to the applied load. The inset illustrates, to scale, the position and the size of both the AFM tip and the X-ray beam during the experiment. FEM simulations of (*b*) the total displacement, (*c*) the stress 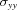 along the wire and (*d*) the volumetric strain for the Au nanowire calculated for a point load of 360 nN using *COMSOL Multiphysics*.

The bending angle measured in the *in situ* µLaue diffraction experiment and the simulated bending angle at a distance of 1.8 µm from the loading point are presented in Fig. 3 ▶(*a*) as a function of the piezo movement 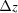. The horizontal error bars originate from the uncertainty of the total piezo movement due to the readout time of the detector and the averaging during the exposure time. The vertical error bars are caused by determining the centre of mass of elongated Laue spots, in particular, for strongly bent wires, thus reducing the accuracy of the local crystallographic unit-cell UB estimation. For the simulated data, 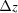 was calculated by summing the deformation of the nanowire in its centre 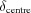 and the necessary deflection of the AFM cantilever, taking into account the applied force *F* and the cantilever stiffness of 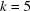
 N m^−1^: 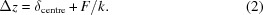



For small wire deformations with a bending angle of less than 1° and thus a displacement in the wire centre of 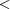
50 nm, *i.e.* less than the half-thickness of the wire, the experimental data are well described employing classical beam theories (Landau & Lifshitz, 1986 ▶). For large wire deflections with bending angles 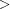
2.5° (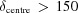
 nm), the bending angle deviates from this classical model and nonlinearities have to be considered. However, the slope of the experimental data decreases more than predicted by the simulations. This discrepancy may be caused by a change of the contact between the AFM tip and the nanowire during mechanical testing and by a lateral force applied as a result of the deflection of the AFM cantilever, which has been shown by a finite bending angle in the 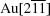 direction (see Fig. 2 ▶ *b*). Moreover, for the computations a thoroughly clamped nanowire was considered, while the wire might slide slightly on its supports.

According to the FEA simulations, the necessary force to obtain the maximum bending angle of 3.5° found in the experiment amounts to 360 nN (corresponding to 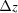 = 72 nm). This force results in a wire deflection of 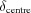 = 190 nm (see Fig. 3 ▶ *b*) and, thus, implies a total piezo movement of 280 nm, which is in very good agreement with the experimental observations. Hence, the elastic behaviour of the nanowire under study can be well described considering bulk values and taking into account geometric nonlinearities. For the maximum load of *F* = 360 nN, the stress 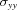 along the long wire axis and the volumetric strain field 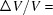 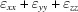 within the nanowire were calculated; these are displayed in Figs. 3 ▶(*c*) and 3 ▶(*d*), respectively.

The maximum calculated stress along the nanowire of 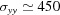
 MPa exceeds the elastic limit of bulk gold by more than two orders of magnitude (Schmid & Boas, 1968 ▶). However, it is still far below the theoretical limit for the material, which is about 4.8 GPa (Wu *et al.*, 2005 ▶) and which has been experimentally demonstrated for ultra-high-strength gold wires (Wu *et al.*, 2005 ▶; Greer & Nix, 2006 ▶; Volkert & Lilleodden, 2006 ▶; Richter *et al.*, 2009 ▶). For 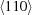-oriented gold nanowires grown by the same method as the ones used in the present work, the elastic limit under tension was found to range from 0.6 to 1.6 GPa (Sedlmayr, 2012 ▶). The computed strain within the nanowire ranges from −0.15 to +0.15%. At the loading point the nanowire exhibits compressive and tensile strains of the same modulus. Owing to the clamping at both wire supports a tensile strain develops along the nanowire, increasing its rigidity. This additional tensile strain, which is not considered in the classical Euler–Bernoulli beam theory, supports the requirement of taking into account geometric nonlinearities. When neglecting terms of higher order, 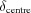 amounts to ∼1.1 µm, which is about a factor of three larger than the total piezo movement during the experiment.

## 4. Conclusions   

The *in situ* three-point bending test of a single Au nanowire in combination with µLaue diffraction presented in this work visualizes the bending process of an individual nanostructure and allows for testing its elastic behaviour. The experimental results can be well described by the classical doubly clamped beam theory, taking into account geometric nonlinearities and using the elastic constants of bulk gold. The elastic limit was found to be at least two orders of magnitude higher than for bulk material, consistent with the literature on ultra-high-strength gold nanowires (Wu *et al.*, 2005 ▶; Richter *et al.*, 2009 ▶). This work is a major step forward in employing Laue diffraction on nanomaterials, paving the way to novel *in situ* nanomechanical studies. For instance, Young’s modulus of a self-suspended nanowire may be determined with higher accuracy by recording the complete wire profile *in situ* instead of monitoring the deformation of one single position only. Moreover, recording the onset of plasticity will allow for studying the nucleation of the first defects and dislocations as well as their evolution and propagation.

## Supplementary Material

Evolution of the complete <it>in situ</it> diffraction patterns. DOI: 10.1107/S1600576715001107/nb5141sup1.avi

Zoom on the two central Laue spots. DOI: 10.1107/S1600576715001107/nb5141sup2.avi

## Footnotes

2 The movies discussed in this paper are available from the IUCr electronic archives (Reference: NB5141).
